# Machine Learning for Online Automatic Prediction of Common Disease Attributes Using Never-Ending Image Learner

**DOI:** 10.3390/diagnostics13010095

**Published:** 2022-12-28

**Authors:** E. Rajesh, Shajahan Basheer, Rajesh Kumar Dhanaraj, Soni Yadav, Seifedine Kadry, Muhammad Attique Khan, Ye Jin Kim, Jae-Hyuk Cha

**Affiliations:** 1 School of Computing Science and Engineering, Galgotias University, Greater Noida 203201, India; 2 Department of Applied Data Science, Noroff University College, 4612 Kristiansand, Norway; 3 Artificial Intelligence Research Center (AIRC), Ajman University, Ajman 346, United Arab Emirates; 4 Department of Electrical and Computer Engineering, Lebanese American University, Byblos P.O. Box 13-5053, Lebanon; 5 Department of Computer Science, HITEC University, Taxila 47080, Pakistan; 6 Department of Computer Science, Hanyang University, Seoul 04763, Republic of Korea

**Keywords:** Internet technology, machine learning, Never-Ending Image Learner, automatic online prediction, online healthcare system, virtual image sensing

## Abstract

The rapid increase in Internet technology and machine-learning devices has opened up new avenues for online healthcare systems. Sometimes, getting medical assistance or healthcare advice online is easier to understand than getting it in person. For mild symptoms, people frequently feel reluctant to visit the hospital or a doctor; instead, they express their questions on numerous healthcare forums. However, predictions may not always be accurate, and there is no assurance that users will always receive a reply to their posts. In addition, some posts are made up, which can misdirect the patient. To address these issues, automatic online prediction (OAP) is proposed. OAP clarifies the idea of employing machine learning to predict the common attributes of disease using Never-Ending Image Learner with an intelligent analysis of disease factors. Never-Ending Image Learner predicts disease factors by selecting from finite data images with minimum structural risk and efficiently predicting efficient real-time images via machine-learning-enabled M-theory. The proposed multi-access edge computing platform works with the machine-learning-assisted automatic prediction from multiple images using multiple-instance learning. Using a Never-Ending Image Learner based on Machine Learning, common disease attributes may be predicted online automatically. This method has deeper storage of images, and their data are stored per the isotropic positioning. The proposed method was compared with existing approaches, such as Multiple-Instance Learning for automated image indexing and hyper-spectrum image classification. Regarding the machine learning of multiple images with the application of isotropic positioning, the operating efficiency is improved, and the results are predicted with better accuracy. In this paper, machine-learning performance metrics for online automatic prediction tools are compiled and compared, and through this survey, the proposed method is shown to achieve higher accuracy, proving its efficiency compared to the existing methods.

## 1. Introduction

Healthcare is one of the most worrisome industries. With the advent of the digital era and technological advantages, many multidimensional data on patients are created, including clinical factors, hospital resources, illness diagnostic information, and medical equipment used. The number of Internet users has been growing exponentially over the years, and there are instances where online healthcare advice is faster to grasp than real-world help. As online health advice is easily obtainable, it can greatly benefit users. Other groups of people leave their responses to the posts of those seeking medical help with predictions of possible diseases. However, these predictions may not always be accurate, and there is no assurance that users will always receive replies to their posts.

Moreover, some posts that are made up can misdirect the patient. Therefore, reliability is a significant issue. According to a survey conducted, it was found that 25% of users lie on social networking sites. A sufficient amount of research on disease prediction has been carried out in recent years. People express their symptoms in non-technical or natural terms, complicating disease prediction. Machine learning is proposed for the automatic online prediction of common disease attributes using Never-Ending Image Learner. NEIL (Never-Ending Image Learner) is a computer program that runs 24 h per day, 7 days per week, to extract visual knowledge from Internet data automatically. This is carried out with the intelligent analysis of disease factors by virtual image sensing. Virtual sensors are a kind of software that provide accessible information and processes and form a pure software abstraction layer without further specifying data-processing aspects. They allow developers to create applications based on motion. Virtual sensors overcome several weaknesses of purely physical sensors, reduce signal noises, and thus increase signal confidence. They obtain data from precisely one physical sensor and mirror. Either may be completely unchanged. They are highly flexible and can be redesigned as required. In this study, machine-learning-assisted automatic prediction from multiple images using multiple-instance learning was performed, and the data were stored as per the isotropic positioning for deeper storage of images and their data. Never-Ending Image Learner was used to predict disease factors, structural risk minimization was used to select from finite image data, and machine-learning-enabled M-theory with virtual sensing for real-time images was employed.

M-theory, known as computational learning theory, aims to understand the fundamental principles of learning as a computational process and combines computer science and statistical tools. A general algorithm for isotropic positioning, the working principle of Never-Ending Image Learner for image prediction, was obtained. The working principle of structural risk minimization, the algorithm used for automatic prediction from datasets, were analyzed. Structural minimization is an inductive principle for model selection used for learning from finite training datasets and is an inductive principle applied in machine learning. The result of the employment of machine-learning-enabled M-theory with virtual sensing is the automatic online prediction of common disease attributes. This article makes the following contributions:

1. Introduces machine-learning-assisted automatic prediction from multiple images using multiple-instance learning;
2. Stores data as per the isotropic positioning for deeper storage of images and their data;
3. Uses Never-Ending Image Learner to predict disease factors;
4. Uses structural risk minimization to select from finite image data;
5. Shows how machine-learning-enabled M-theory with virtual sensing can be used to predict real-time images.

This research focused on Machine Learning for automatic online prediction of disease-common attributes using Never Ending Image Learner. The Smart analysis of disease factors with virtual image sensing was performed using Machine Learning, Never Ending Image Learning, and Virtual image sensing Techniques. This research illustrates 2 Proposed System parts under a Multi-access edge computing Platform. In particular, the analysis of automatic online prediction explains Machine Learning assisted automatic prediction from multiple images using Multiple instance learning methods very clearly. Store data from this description can be used to verify their data using more profound storage images based on the “Isotropic position” method. And the Never Ending Image Learner of disease standard attribute prediction proposed here has been designed with the three things given below, and their efficiency has been tried to show the given problem formulation very correctly respectively.

- Forming the “Never Ending Image Learner” for predicting the disease factors
- Forming the “Structural risk minimization” for selecting from finite data images.
- Machine Learning Enabled M-Theory with Virtual sensing for predicting the real-time images

All three methods mentioned above not only accurately complete the automatic online prediction of disease-common attributes but also handle the probabilities that may occur in them in the following manner and use the Multiple instance learning methods to get the best output. The Experimental Results and Discussion section (Section 4) presents the probabilistic results taken here.

The article’s organization is as follows: Section 1 describes Machine Learning for Online Automatic Prediction of Common Disease Attributes Using Never-Ending Image Learners with their respective outcomes and highlights the contribution. Section 2 describes the literature review on machine learning and automatic production techniques. Section 3 describes proposed methodologies like Ontology Mapping for Healthcare and respective Structural Risk Minimization on the Common Disease. Section 4 describes the result and the relevant result concerning Machine learning for automatic online prediction. Finally, the research ended with a Conclusion and the respective references.

## 2. Related Work

Various automatic diagnostic models are used to identify diseases in their early stages. Machine-learning models are effective ways of diagnosing diseases. This work collects data via an Android app, and a machine-learning algorithm is applied to real-time data collected with the mobile phone. For early detection and prediction, the logistic regression method is used to identify the type of disease, and the treatments for the disease are given as outputs by implementing the comparative analysis proposed in [1].

A diagnosis is made using the decision support system. Text is mined from different IoT devices, and the decision-making process to identify the disease is implemented. QMR, MYCIN, Iliad, Intern-ist-I, DXplain, Isabel, and Baidu’s “medical robot” are used for the clinical decision identifying the disease. Probability computation and symptom accuracy are needed for evaluation in a matrix format, and the disease is determined. In addition, this paper proposes a means of intimating the side effects of all the drugs pre-installed in the dataset [2]. This decision support system support has to make our proposed system concerning automatic online prediction. This automatic online prediction greatly helps make disease-common attribute predictions using Machine Learning.

Analyze validation and the execution purpose, and machine learning is used to analyze the data, according to [3]. A machine-learning algorithm is used to diagnose the disease, describing the disease and its stages. The disease is classified and predicted using the predefined dataset for the symptoms, side effects, diseases related to the symptoms, etc. Therefore, data processing and machine learning are the instruments for disease diagnosis [3]. Their machine-learning algorithm supports us in making Machine Learning for Smart analysis of disease factors. This [3] system clarifies some inquiries on disease diagnosis.

The Never-Ending Image Learner (NEIL) runs automatically 24/7 with access to Internet data. This produces a similar relationship for the in-stances or a label that categorizes the knowledge of the labeling effects. The micro- and macro-vision deliver outputs to understand the sense relation, similar to the image and the pieces of information. NEIL invents 400 K instance visuals and 1700 relations. This was suggested in [4]. There Never-Ending Image Learner (NEIL) supports us in making and predicting the disease factors. Based on this Never-Ending Image Learner (NEIL), sense relations are determined successfully [4].

The use of supported living and intelligent monitoring technologies to evaluate the cognitive health of young autistic people is addressed in [5]. They assessed the emotions and cognitive abilities of autistic young people without using medical interventions [5]. With the use of intelligent-systems-based artificial intelligence (AI) and Internet of Things (IoT) technologies, this research enabled us to enhance the cognitive abilities of autistic people. The suggested IoT system with AI capabilities helps with the research on the present application’s heart rate prediction function. Additionally, this tool fosters social communication skills and offers a new virtual environment to support young people online. Intelligent monitoring technologies help us improve disease-common attribute prediction.

The diagnosis and management of cardiovascular disease (CVD)-related sickness and demise were covered in [6]. Cardiology uses artificial intelligence (AI) technologies to improve and optimize CVD outcomes. The data’s complexity is decreased through optimization and machine learning [6]. This boosting procedure increases data volume and complexity to improve optimization and therapeutically beneficial information retrieval based on machine learning. This extraction procedure uses machine learning to treat enormous numbers of medical data. The optimization machine learning procedural aspect supports busting data volume processing and reducing the complexity of diagnosis and management of any disease. These upgrades are implemented in daily life. From this angle, the feature selection in our research was optimized using the meta-heuristic algorithm classification.

Patients’ radiograph chest X-ray images are used to detect viral attacks. With accurate classification, coronavirus infection is determined with 99.7% accuracy. The performance is achieved based on the dataset collected from the human chest. The deep-transfer-learning approaches for identifying coronavirus disease in its early stages are elaborated. This AAP ticket support has to make an apparent classification rate and high accuracy rate [7]. This research encourages proposed research efficiently. This performance of existing related research helps to make a successful creation of Machine Learning for automatic online prediction of disease common attributes using Never Ending Image Learner by Smart analysis of disease factors with virtual image sensing.

## 3. Methods

The massive adaptation of computer-based technology in the healthcare industry helps to accumulate electronic data. However, due to many medical records, doctors have difficulty accurately identifying diseases from presented symptoms sufficiently early to prevent patients from developing severe conditions. Significant research is being conducted to predict disease via the latest technologies. The most common method is gathering disease images and processing the collected images so that the disease can be predicted with the help of machine-learning technologies. Such methods are increasingly used to help patients, and medical practitioners overcome the challenges of diagnosing disease. These methods can be found in modern healthcare systems’ decision-making processes [8].

The proposed system aims to find the disease road map via the patient’s common attributes, which might be gathered through a physical examination or by remote devices that collect data from patients found in the database. The machine-learning method automatically predicts diseases with typical symptoms or attributes using NEIL (Never-Ending Image Learner). This program is used to learn from the information in an image based on the relationships between the objects in the image and predict the factors of diseases. However, isotropic positioning is the probability distribution over the vectors used for more profound image storage concerning the random matrix. To determine disease road maps, this research uses the ontology healthcare system to analyze the disease path based on symptom factors. Machine-learning-enabled M-theory with virtual sensing makes predictions from real-time images [9].

### 3.1. Ontology Mapping for Healthcare

Ontology mapping is essential in medicine in describing medical terminological concepts and finding relations by adopting medical knowledge in clinical practices. Ontology mapping aims to provide an exhaustive and definitive classification of the relevant entities, i.e., it can provide answers to questions and exhaustively represents the entire class of entity types that should be included in the classification process, also based on the relations of diseases according to their types.

**Figure 1:**
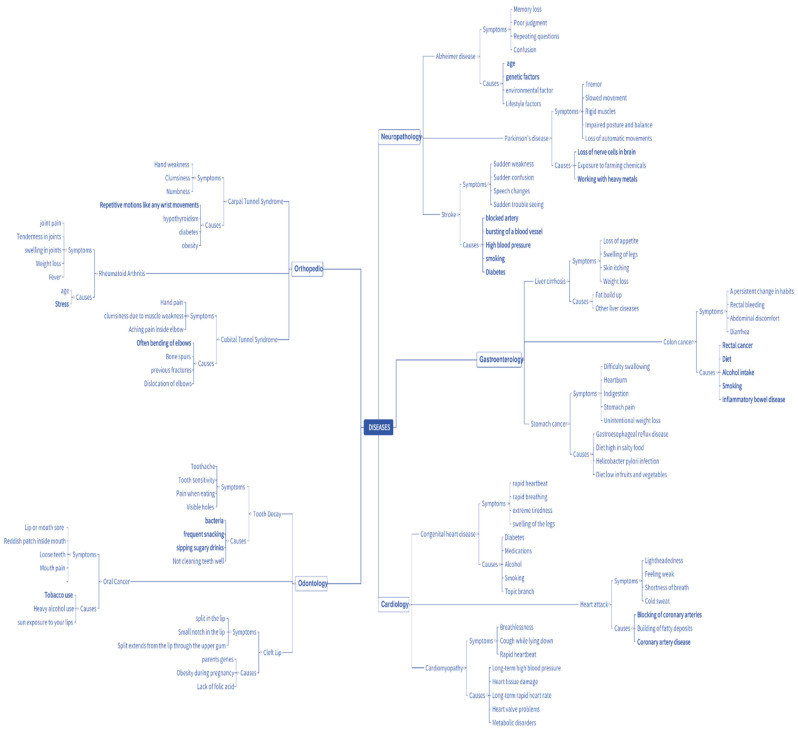
Flow diagram of the prediction of common disease attributes.

In this system, the complete structure of the ontology is determined based on diseases and their attributes, such as symptoms, causes, etc. [10]. A disease is considered a psychological or physiological dysfunction for which symptoms are attributes of the disease which occur frequently. Each disease might have different symptoms, and there is a chance of two or more diseases having similar symptoms. In this system, the ontology is constructed to find the common symptoms to be factored into identifying the disease and represented in a tree structure. A tree-structure-based ontology mapping represents the healthcare disease roadmap. This model was inspired based on the protégé system software tool representation [11]. This model was applied to the ML-assisted automatic prediction, and it was proposed to predict the comment attributes with the help of the ontology tree-level structure (Figure 1).

### 3.2. ML-Assisted Automatic Prediction

Machine learning (ML) was used to develop the algorithm for improving the automation process with experience and use of any data, and the model building was based on the training data for the prediction and decision-making process. ML can be used advantageously in healthcare, as the considerable numbers of medical data reduce ML’s burden with predicting data [12]. The proposed system uses ML for the automatic prediction of common disease factors by inputting image data through a process of analysis; thus, for the process of prediction, the ML algorithm needs to have the ability to learn by itself based on the training dataset provided for the accurate prediction of the common attributes of diseases.

For automatic data prediction, images are inputs for extracting common attributes. Having been provided as the input, the disease image then undergoes conversion to obtain data for extracting predicted symptoms from the ontology structure. The image is converted by comparing the input with the historical disease database, which contains all types of diseases with their names; thus, by comparison, the particular disease can be identified by image-processing technology for the extraction of the features of the image if the feature match with the historical data and the name of the disease is taken for processing. Then, the converted data are taken as inputs to extract the common attributes of the disease [13]. Hence, this extraction training dataset plays an important role, so the ML-embedded multiple-instance learning (MLeMIL) process was considered for practical training of the system with the datasets.

Training of the algorithm using a particular learning model is performed since both learning strategies respond to data collection, and motivation is distinctively present. Similar to machine learning is supervised machine learning. This type of machine learning is divided into supervised, unsupervised, and reinforcement learning to give the unlabeled data needed to analyze and uncover data. These methods are used on neural networks for grouping and anomaly identification. On the other hand, supervised machine learning is helpful in data science in looking for unidentified similarities and contrasts in data. While the data are being processed, the dimensions of the related groups are also decreased [14,15].

In machine learning (MLeMIL), multiple-instance learning is supervised learning. This process involves the receipt of a set of instances. These are labeled individually, and the learner receives a set of labeled bags containing many instances. In ML-embedded multiple-instance learning (MLeMIL), a single class label is provided for a set of instances. This method was adopted with machine learning (ML) to predict the common attributes of disease using the automatic prediction method by considering multiple inputted images. The multiple-image datasets are acquired with the help of the virtual sensing method, where different sensors are used to obtain data from multiple points simultaneously, which are stored in databases for later processing according to the structure of the ontology.

**Figure 2:**
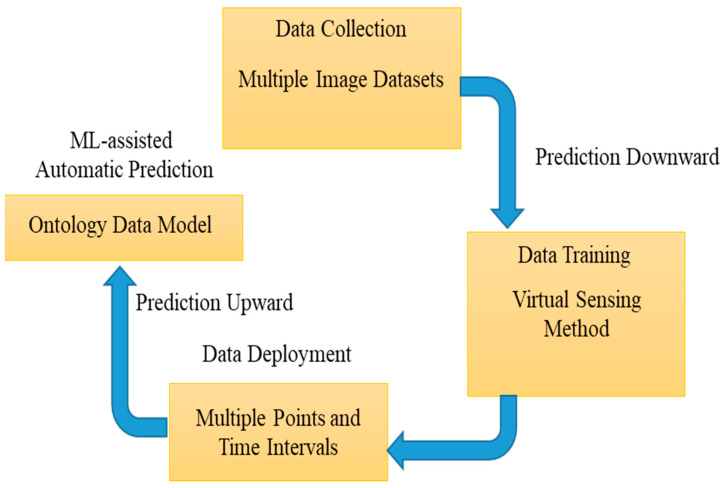
ML-embedded multiple-instance learning (MLeMIL) process flow.

Figure 2 shows the ML-embedded multiple-instance learning (MLeMIL) process flow per the predicted measured data. The measured data are mapped based on the disease symptoms’ clinical metrics; hence, the traditional discriminative learning algorithm is used for training. A binary classifier P(B|A) is used for the estimation of the required training dataset of the form {($A_{1},\;B_{1}$), …, ($A_{n},\;B_{n}$)}, where $A_{i}$ is determined as the instances and $B_{i}$ is considered as the binary label, represented as $B_{i}\;\epsilon \;\left\{0,1\right\}$. The framework for training data in multiple-instance learning is of the form {($A_{1},\;B_{1}$), …, ($A_{n},\;B_{n}$)}, where the bag $A_{i}$ and $B_{i}$ are the labels on the bag. This is stated mathematically in Equation (1):

(1) $$B_{i}=\max j\left(B_{ij}\right)$$

where $B_{ij}$ are the labels of instances assumed to exist but unknown during the training process. In other words, the bag is determined as positive only when it consists of only one positive instance, and many algorithms are used to solve the problems of MLeMIL in this system. Machine learning with MLeMIL is proposed, and it uses the framework of gradient boosting to train the classifier, which reduces the bag log-likelihood determined as in Equation (2):

(2) $$\log\;l=\sum_{i}\left(\log P(B_{i}|a_{i})\right)$$

This states that the likelihood concerning the bags is determined and not the instances, as the labels of instances are not known at the period of training, and the main goal of the process is to train the classifier of instances, estimated as *P*(*B*|*A*); then, the probability of a bag is determined as positive concerning the instances, expressed as *P*($B_{i}$|$A_{i}$), and the NOR model is adopted. The process is then as follows in Equation (3):

(3) $$P\left(B_{i}|a_{i}\right)=1-\prod_{j}\left(1-P(B_{i}|A_{ij}\right))$$

The above equation states that the bag consists of the required properties when one of the instances in the bag has the highest probability rate, and then the probability of the bag will also be higher. In the proposed system, instance-based learning with ML involves a set of learning algorithms that help to perform predictions without explicitly performing generalizations. New instances are compared with training instances stored in the memory to find the common disease attributes [16].

As shown in Figure 2, our proposed algorithm initially computes the similarity of all attributes in an image. GO disease data are determined for each attribute in the image based on the other attributes with the disease data. The training set of disease data is labeled disease data (i, a), showing that attribute a has disease datum i. The similarity graphs indicate the integrated multiple sources to predict whether an attribute receives a particular disease datum [17]. This process is based on the prediction algorithm. Outputs of −1 and +1 represent the similarity of an attribute: −1 represents high dissimilarity between the attributes in one image and those in another; similarly, +1 indicates high similarity between the two images. From these determinations, the GO dataset’s automatic prediction is performed by the following algorithm [18].

ML-assisted automatic prediction refers to the output of an algorithm after it has been trained on a historical dataset and applied to new data when forecasting the likelihood of a particular outcome. In the present study, ML-assisted automatic prediction of patient health data-handling performance has been presented. The central part of this research focuses on applications of ML-assisted automatic prediction of health data handling and its prediction. To this end, previous research works which investigated the application of ML and prediction characterization have been reviewed and discussed [19]. ML-assisted automatic prediction was used to assess image learning to further this aim. It was reported that Never-Ending Image Learner used online healthcare system management.

**Figure 3:**
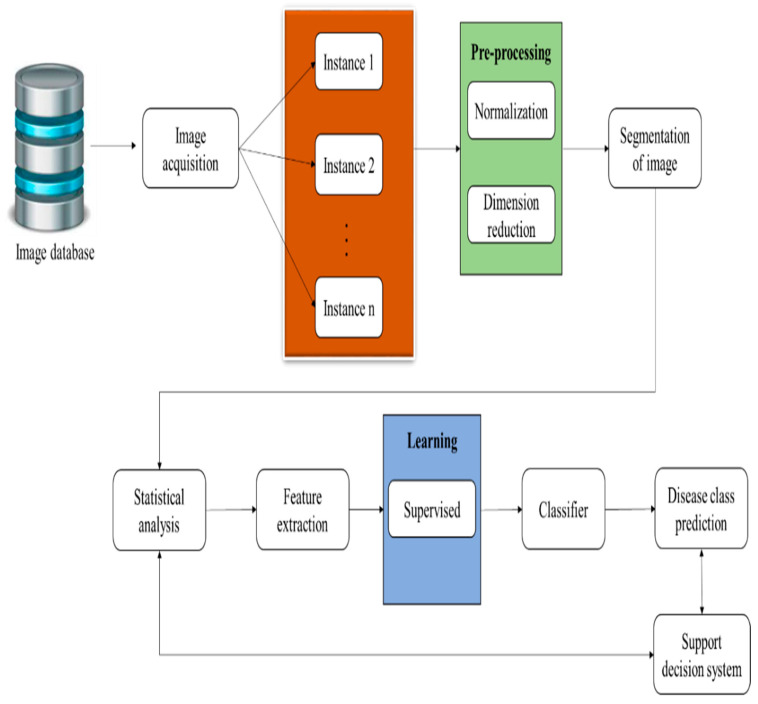
Machine-learning-assisted automatic prediction using multiple-instance learning.

Some of the standard ML-assisted autonomous health data prediction algorithms are shown in Figure 1. In machine learning, the algorithm learns from labeled training data to assist in forecasting health data results, while the program uses preprocessed data to find associations between characteristics of interest. Figure 3 illustrates frequently used ML prediction techniques for addressing various virtual image-sensing challenges. Using real-time data in offline mode contributes to maximizing real-time prediction and maintaining the minimal latency in machine learning: this minimum delay performance and prediction delay must be balanced with the ML-aided autonomous prediction. ML models demand both synchronous and asynchronously predicted sequence data. This aids in recovering the prediction outcome from the ML optimization against the forecast of low-read latency [4]. Last but not least, this ML-assisted automatic prediction aids in removing unnecessary, pointless, or irrelevant data to support the prediction mode using static reference features and dynamic real-time features.

As per Algorithm 1, for each image dataset, the function F; AxA→[0,1], F denotes the similarity function, where A is a set of attributes. The similarity between an attribute in two images is defined as the Pearson correlation coefficient associated with an array of two attributes in Equation (4):

(4) $$R(x,\alpha )=\frac{\sum \left(x_{k}-\bar{x}\right)\left(\alpha_{k}-\bar{\alpha }\right)}{\sum [{\left(x_{k}-\bar{x}{)}^{2}\sum {\left(\alpha_{k}-\bar{\alpha }\right)}^{2}\right]}^{1/2}}$$

where $x_{k}$ shows the element from the *x* array and $\bar{x}$ denotes the mean of array element *x*; similarly, $\alpha_{k}$ shows the element from the α array and $\bar{\alpha }$ Denotes the mean of array element α. Then, the graph for the weight of the attribute edge and the vertices of an attribute is constructed. The weight of the edge is calculated in two ways: the first method involves adding a value of similarity between the two attributes; the second method involves building a graph by adding the similarity functions to find the weights. In the graph, each node represents an attribute, and the weight of the edge represents the similarity between the attributes. An attribute is removed from the graph for each disease datum (i, t), and the threshold value is calculated. The similarity threshold value is low when an attribute t with disease datum I has lesser total similarity than another attribute with this disease datum. The upper threshold is calculated by t without I, the most similar attribute with a disease datum.
**Algorithm 1.** Automatic prediction from the dataset.input; dataset output; prediction of attributes for each disease data i           for each attribute a                      remove → a                     for each disease data (i, t)                                MIN = 0                                for each disease data (i, k) & (k≠t)                                          MIN = MIN + S (k, t)                                          if (MIN < low_Thold)                                                    low_Thold = MIN                                          end if                                end for                      end for                      for each disease data (i, t)                                MAX = 0                                for each disease data (i, k) & (k≠t)                                          MAX = MAX + S (k, t)                                                     if (MAX > high_Thold)                                                              high_Thold = MAX                                                              add → a                                                              Ts = 0                                                    end if                                end for                      end for                      for each (i, t) & (a≠t)                                Ts = Ts + S (a, t)                      end for                      if (Ts > high_Thold)                                predict disease data (i, a)                                else if (Ts > low_Thold)                                          interpolated S = interpolate (Ts, low_Thold, high_Thold)                      end if                       if (interpolated S > cut-off)                                predict disease data (i, a)                      end if            end for end for 

The process predicts whether attributes ought to be instructed with disease datum i. This process includes four steps. First, low_Thold calculates the lowest similarity for an attribute. Next, high_Thold calculates the highest similarity for an attribute [20]. Then, the total similarity (*T_s_*) of all attributes known to have a is computed. If *T_s_* exceeds high_Thold, then a is predicted to have disease datum i. If *T_s_* is less than low_Thold, then a is not predicted to have disease datum i. Finally, the prediction is constructed. If total_similarity is between low_Thold and high_Thold, it is interpolated between the two thresholds to give a number between 0 and 1 in Equation (5):

(5) $$\mathrm{interpolated}\_S=\frac{T_{s}-\mathrm{low}\_\mathrm{Thold}}{\mathrm{high}\_\mathrm{Thold}-\mathrm{low}\_\mathrm{Thold}}$$

A predefined cut-off is 0.5; this is then used to determine whether or not to assign the disease datum to attribute a. Thus, if the cut-off = 0.5 and interpolated_S = 0.6 for attribute a and disease datum I, then attribute a would be predicted to have disease datum i.

**Figure 4:**
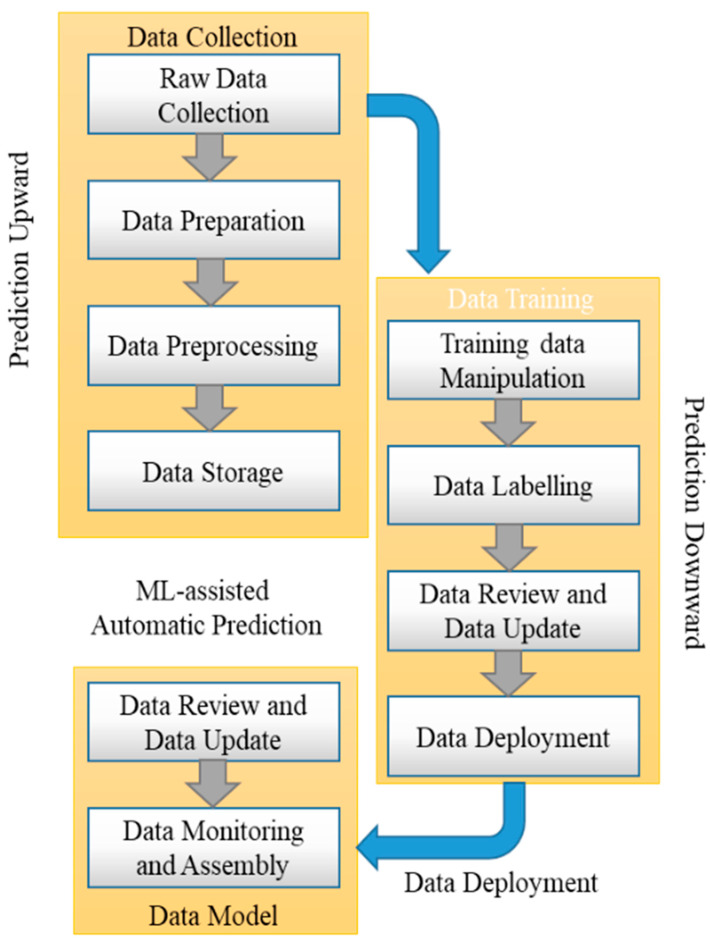
ML-assisted automatic prediction of health data.

**Figure 5:**
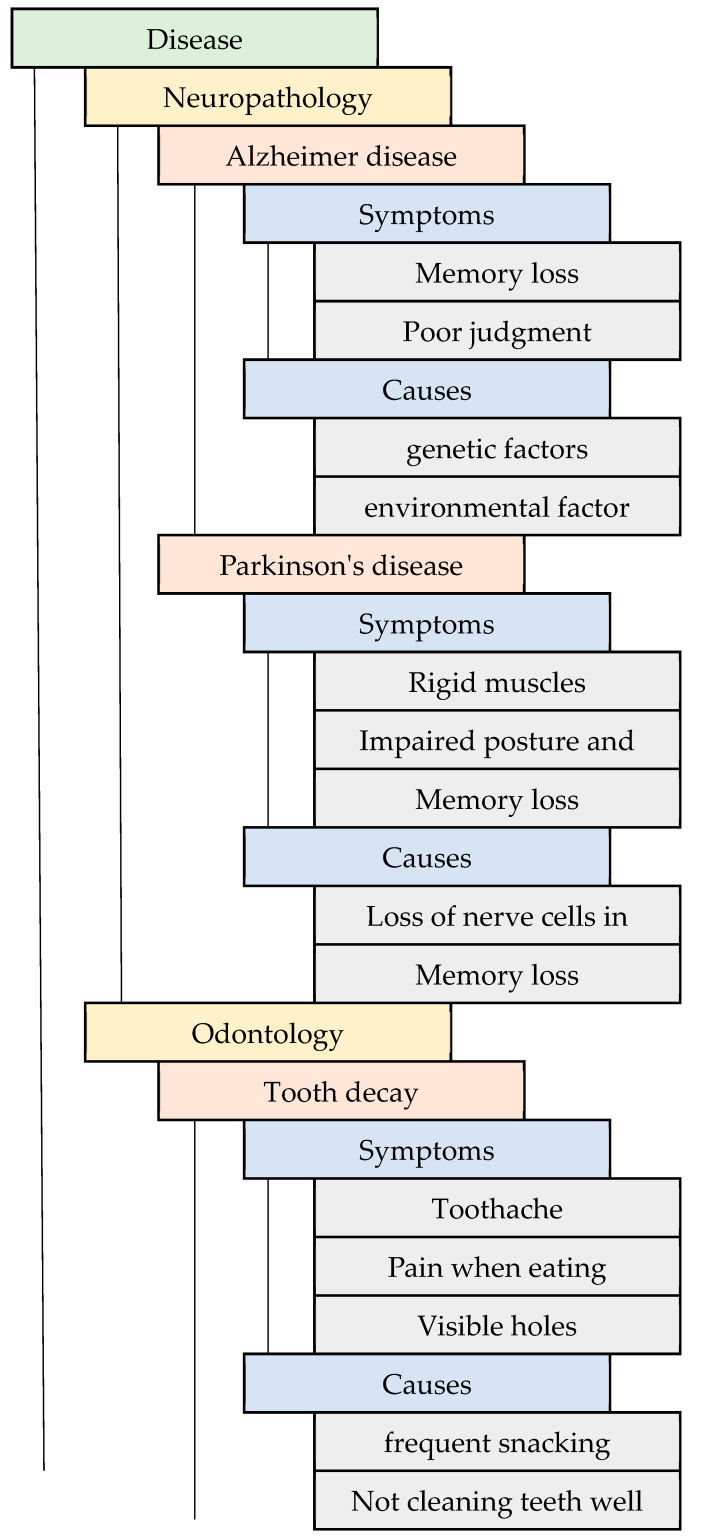
Ontology mapping with a tree-level structure.

After applying the above automatic prediction, the prediction structure for the disease’s common attributes is instantaneously described based on the following ontology structure. This structure, used to predict the common attributes of diseases, is shown in Figure 4. Typically, the structure of an ontology network is enormous. It is infinite; hence, the prediction of common attributes of a disease is an enormous process. It includes the patient’s condition when they met the doctor for the first time but shows how the disease is related to the attributes considered in Figure 5. The disease is the class and contains sub-classes, such as neuropathology, cardiology, orthopedics, and so on, which are fields of disease in which there may be different diseases, for instance, diseases such as Parkinson’s, Alzheimer’s, etc., may constitute a sub-class. Each disease has different symptoms and causes based on its characteristics.

In this research, which aims to find the symptoms of diseases, the ontology structure ends with the instance symptoms and causes, but usually, ontologies grow more extensive, especially with medical data. Ontology analysis is based on the representation of tabulations concerning the tables embedded within the text, thus forming table-based classes and individuals [21]. The ontology table helps identify relationships and the dependencies between entities; then, the verification of dependencies should be checked if the disease depends on the symptoms.

Ontology mapping with a tree-level structure is represented in Figure 5. The disease is the superclass and has the sub-classes neuropathology, odontology, and so on; the attributes of the disease are also provided in the table, which helps in the effortless analysis of the data for the prediction process. To examine the condition of the actual prediction process from the ontology mapping with the tree-level structure, an example training image (a tooth image) is taken and embedded in the isotropic positioning process [22].

### 3.3. Isotropic Positioning

**Figure 6:**
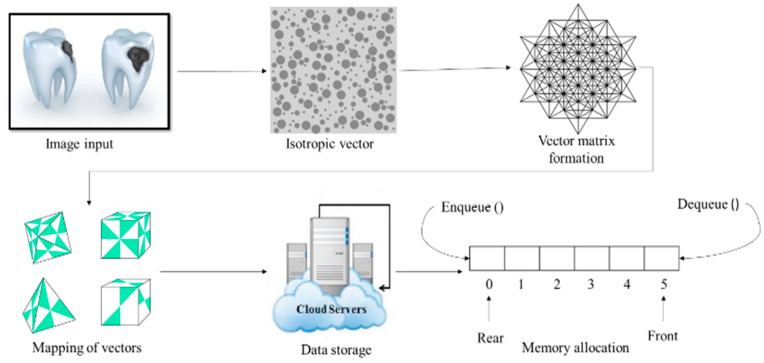
Isotropic positioning for deeper storage of images and their data.

An isotropic group consists of an isomorphism from any datum to itself in a groupoid. The isotropic position is a probability distribution over a vector space if its covariance matrix is the identity matrix (Figure 6).

This isotropic positioning works based on the following algorithm for fine-tuning positional data from an image as per Algorithm 2.
**Algorithm 2.** Isotropic positioning.input; d-distribution ⊂RN, V-vector for Vϵ d          if (EVVT = ID)                     if (uniform distribution over ID)                               V of an orthogonal set ← isotropic                     end if          end if          for all b RN                     if (k ϵ RN)                               if(|k| = 1) & (β > 0)                                         $\int {\langle a,b\rangle }^{2}dx=\beta^{2}{\left|b\right|}^{2}$
                               end if                     end if          end for end for return, Isotropic position for storing data

The above algorithm, d, shows a probability distribution over the RN vector field from the above algorithm. If d is in the isotropic position, then, for each vector sampled (V), the distribution is as expressed in Equation (6):

EVVT = ID(6)

The set of vectors is called an isotropic position when the uniform distribution is over the isotropic position. For every set of orthonormal vectors, the vectors are isotropic for each vector b in RN when the convex body has the volume |k| = 1 center of weight at the origin and constant β > 0, as in Equation (7):

(7) $$\int {\langle a,b\rangle }^{2}dx=\beta^{2}{\left|b\right|}^{2}$$

After amplifying the image’s potential gains in positional data, structural risk minimization is employed to obtain better isotropic positioning and prediction results. Structural risk minimization is fleshed out in the following structural representations [23].

### 3.4. Structural Risk Minimization

Risk is reduced by altering or reducing the number of data used for processing and minimizing the overall processing time. Medical records contain large numbers of data, and the structure of an ontology grows as the number of data tends to infinity; thus, this is considered the biggest challenge in predicting disease attributes. Given the unlimited data in the system, the ontology structure begins with the patient and ends with the condition of a patient at the end of the treatment; the type of disease and its symptoms are found to be part of the ontology. Thus, when input is provided to find the disease’s common symptoms, the prediction process begins from the head of the ontology and traverses the entire network of nodes in the ontology structure. This prediction takes time; hence, finding a finite number of data is preferred to avoid time-consuming and difficult predictions. The data collected from the different sources will be stored in the database. Thus, to predict the common disease attributes, data mining helps determine the patterns from the data source.

Various working principles help handle structural risk minimization concerning training and testing with data samples per Algorithm 3.
**Algorithm 3.** Working principles of structural risk minimization.input; image data—{(a1, b1)…, (at, bt)}, f(a)-function, E(f) = expected risk function, generate expected risk function E(f) = $\int Q\left(b,f\left(a\right)\right)dq\left(a,b\right)$
for unknown problem          find empirical risk function          E(f) ← Eemp(f)          Eemp(f) = 1/t$\sum_{k=1}^{t}Q\left(b_{k},f\left(a_{k}\right)\right)$          for nonnegative set                     if (0 ≤ Q (b, f(a)) ≤ Y)                                P ← 1− η                                else if (Q (b, f(a)) ≤ Y)                                E(f) ≤ Eemp(f) + $\frac{Y\zeta }{2}(\sqrt{1+\frac{4E_{emp\;}\left(f\right)}{Y\zeta }})$                      end if                       ζ ← 2(ln n-ln η)/L                      if L/d large                                Eemp(f) ← small                                E(f) ← small                      end if           end for end for return, minimization of structural risk

The inputs of the distributed samples {(a1, b1)…, (at, bt)} are used to find the function that maximizes the expected function, as in Equation (8):

(8) $$E(f)=\int Q\left(b,f\left(a\right)\right)dq\left(a,b\right)$$

where $q\left(a,b\right)$ represents the joint probability and $Q\left(b,f\left(a\right)\right)$ shows an error of a loss function. The empirical result is shown below and is replaced with an expected function. The function and values *b* and *q* (*a*, *b*) are unknown (Equation (9)).

(9) $$\mathrm{Eemp}(f)=1/t\sum_{k=1}^{t}Q\left(b_{k},f\left(a_{k}\right)\right)$$

For a nonnegative set F, 0 ≤ Q (b, f(a)) ≤ Y follows the inequality that holds the inequality and the probability 1-η reached simultaneously for all loss functions (Q (b, f(a)) ≤ Y), as in Equation (10):

(10) $$E(f)\;\leq \;\mathrm{Eemp}(f)+\frac{Y\zeta }{2}(\sqrt{1+\frac{4E_{emp\;}\left(f\right)}{Y\zeta }})$$

Here, the function has a finite number of dimensions (d) and an unlimited number of elements. d denotes the complexity of a function, and *ζ* is expressed as, First, sum the right of the empirical risk errors seen in the objects; second, sum the interval of confidence that depends on the complexity:

(11) *ζ* ← 4(d ln (2L/d) + d) − ln(η/4))/L

where L denotes the size of the sample. In this case, the function $Q(b,f\left(a\right)$ has a finite number of elements (n), and ζ is expressed as:

(12) *ζ* ← 2(ln n − ln η)/L

These two considerations are followed simultaneously. If L/d seems large, the second sum on the right side tends to be small. Then, the empirical result is negligible. Similarly, the expected value is small (in Equations (11) and (12)). Similarly, if L/d seems small, the second sum on the right side tends to be significant. Then, the empirical result is not responsible for obtaining an excellent or generalized expected value.

Structural risk minimization is intended to reduce the operational risk based on the empirical complexity of the objects. Each set function has a nested subset of a function, where each element must satisfy the following properties. Let S be the function, and the dimension dk of each set of functions be finite, d1 ≤ d2… ≤ dn……, with each element in a set structure Sk having a boundary: 0 ≤ *Q* (*b*, *f*(*a*)) ≤ Y, f ϵ F. The nested structure is constructed concerning complexity. Then, the subset is selected to minimize the empirical risk of deriving the bound of the expected value. When element S and dimension d are selected, the sum of the empirical risk and confidence interval, namely, the expected risk, is minimized [17]. Structural risk minimization suggests a trade-off between complexity and empirical risk. As the subset index increases, the empirical risk will decrease because of the increased inability to approximate the objects when the confidence interval increases based on increased complexity.

**Figure 7:**
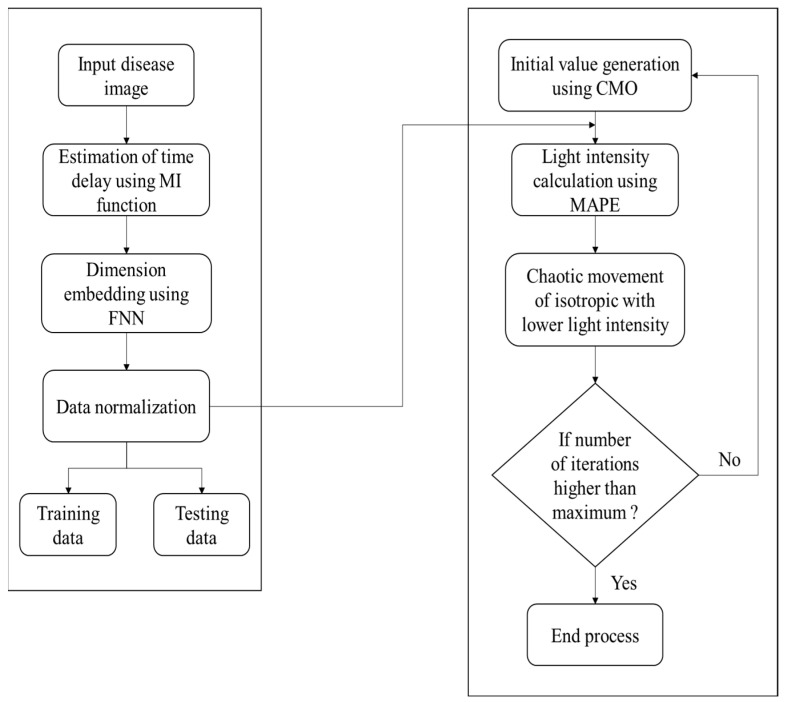
Structural risk minimization for finite data.

Structural risk minimization was adopted with never-ending learning for learning unknown sources to relate to the existing available data sources [24]. Only the disease and its attributes are required to predict the common attributes; the process begins with the class disease and traverses the ontology network to find the attributes faster and more effectively. Figure 7 represents structural risk minimization for finite data based on the predicted data from the ontology.

### 3.5. NEL (Never-Ending Learning)

Never-ending learning is a machine-learning system that develops answers to the questions that users pose in natural language without the intervention of humans and can learn continuously based on the relationships between objects. NEL adapts the self-correction process when it contains more information with the help of machine learning [25]. It was developed to identify the fundamental semantic relationships between the numbers of predefined data categories [26]. Recently, the number of relationships available in the knowledge base of NEL has increased, and we have learned more new facts. Thus, the research team has been running NEL around the clock, sifting through large numbers of web pages looking for connections between items of information which are already known, and, through the process of search, trying to make new connections; thus, in this way, it represents the learning strategy of humans.

**Figure 8:**
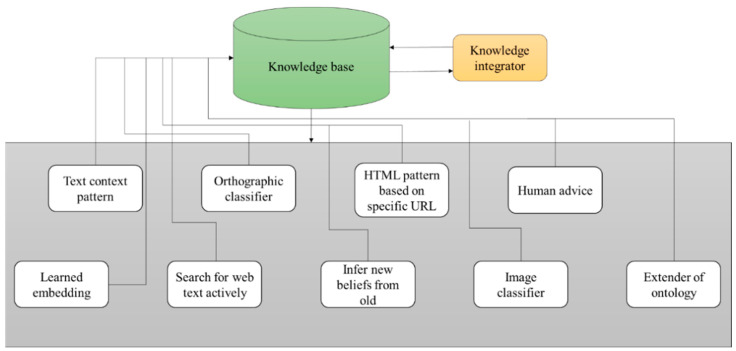
Never-Ending Learning Architecture.

The architecture of never-ending learning is shown in Figure 8. It consists of a knowledge base with a knowledge integrator, which means merging two or more originally unrelated knowledge structures into a single structure. It also consists of patterns of text, orthographic classifiers, HTML patterns based on specific URLs, human advice, learned embedding, active searching of text on the web, inferring new beliefs from old ones, image classifiers, and ontology extenders [27].

**Figure 9:**
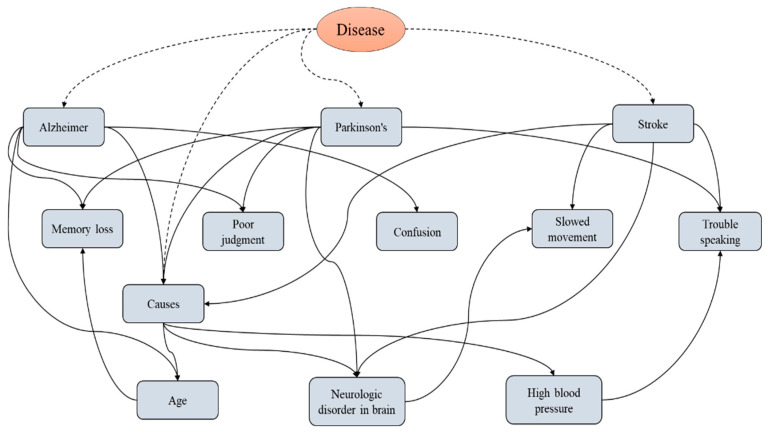
Never-ending learning for predicting similarity factors of disease.

From Figure 9 and the consideration of the never-ending learning architecture, the never-ending learning method, represented in the following algorithm, was used to predict similarity factors in images of tooth disease [28].

As per Algorithm 4, Never-Ending Image Learner for image prediction, the sample outcome is predicted per the disease and its symptoms [29].
**Algorithm 4.** Never-Ending Image Learner for image prediction.**input;** an ontology-O **output;** trusted instances for each group share initial image data **for** k = 1, 2…∞            for each group ϵ O                     extract new image data                      filter patients                     train data classifiers                     assess the patient using a trained classifier                      promote highest-confidence patient           end for           Share items **end for**
**for** each class           **if** X mutually exclusive with Y                     Y ← negative instance of image           **end if**          **if** (Y(X) ← X)                      Y ← trusted item           **end if**          if (co-occur ← two trusted patterns) & (co-occur ← any –Ve pattern in the same web)                      NELL then filters out           end if end for

This framework predicts a disease’s common attributes, taking symptoms and relevant parameters as inputs. Text is taken as input based on the names of the symptoms related to the particular disease. In addition, the other parameters help to identify the disease accurately. Sometimes several diseases have the same symptoms; we can filter some diseases with this method. Then, by analyzing other parameters, we can accurately identify the disease and determine a common disease attribute. Creating a database with five user input parameters helps to identify the common disease attributes. Table 1, below, is based on automatic online prediction concerning common disease attributes in ontology mapping. Finally, the process ends with the automatic online prediction of common disease attributes based on the following ontology mapping tree.

In this system for automatic online prediction of common disease attributes, the general contents of a database include the name of a symptom, the time, intensity, the name of the organ, and the disease duration. As per Algorithm 5, a word-tagging process is implemented to connect the text with respective symptoms and another parameter. This process follows three technics [30].
**Algorithm 5.** Automatic online prediction of common disease attributes.generate database-DB = {symptom, time, intensity, organ name, duration} create a tag for symptom user input as word **for** each user word          separate each word           check each word in DB           **if** (word → found)                     put relevant array                     **else if** (word → not found)                      Search symptoms and reference tag                     **if** (reference word → found)                                move to the relevant decision                               **else**                                         put relative attributes and continue                     **end if**          **end if** **end for** **return**, prediction of common disease attribute

6. Synonym parent tree

When the input text is matched with the correct database, many results are unmatched, even if the word’s meaning is the same. In this case, the synonym parent tree is used. Each word is connected with the root word. If there is any matching child word, the input word is replaced with the root of the matched word [13].

7. Symptom reference tag

When a particular symptom is referred to with a problematic one-word clinical term, the user may use a word in many ways, such that identifying the referent may be tricky. A symptom reference tag method that determines the name of the symptom using compound words was therefore introduced.

8. Relevant attribute array

The disease can be identified with typical symptoms based on the above process. If the symptom input is in textual form, the relevant array form of an input symptom is taken. Similarly, if the input shows a time, it modifies and enters the relevant time array.

## 4. Experimental Results and Discussion

Machine learning is the study of computer algorithms that can automatically improve through experience by using data. Machine learning is used to process vast numbers of images, which requires processing vast numbers of data, often of high dimensions, which is problematic for most machine-learning techniques. Therefore, interactions between image data and priors are necessary to drive model-selection strategies. Never-Ending Image Learner (NEIL) is a computer program that can learn information about images found on the internet. Virtual image sensing involves an electronic device that converts an optical image into an electronic signal. It is used in digital cameras and imaging devices to convert the light received by the camera or imaging device lens into a digital image [31].

### 4.1. Overall Accuracy

The proposed system focuses on machine learning for automatic online prediction of common disease attributes using Never-Ending Image Learner. The search result provides overall accuracy, image-sensing accuracy, error standard deviation, normalized frequency, and image classification and has a prediction function. This analysis’s approximate result is 52.20%.

A detailed explanation of an image provides textual access to visual content, which is most often used for digital graphics online and digital files. Table 2 shows the sample image and overall accuracies based on the classification. This graphical representation is based on the sample images’ true-positive, true-negative, positive, negative, and overall accuracy. Overall accuracy is the probability that a test will correctly classify an individual. The sum of the true positives plus the true negatives is divided by the total number of individuals tested [32]. For the sample images and the overall accuracy based on the classification inference results, the sample image count level was 25 to 950, and the increased accuracy range was 97.8%. Image accuracy is the distance between the actual geographic location of an object or detail compared to the object’s position in the image. Table 2 shows the clustering based on the number of image samples and the image-sensing accuracy, and an error matrix is the most common way of expressing the accuracy of remote-sensing image classifications, such as the smart analysis of disease factors with virtual image sensing. The clustering is based on the number of image samples and image-sensing accuracy inference results. The image sample counts are 10 to 262, and the increased image-sensing accuracy range is 65.34%.

**Figure 10:**
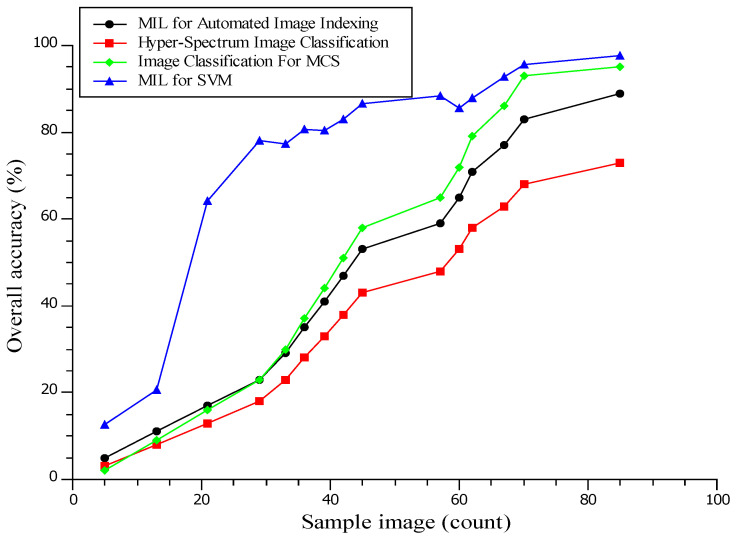
Analysis of Overall Classification Accuracy.

In Figure 10, MIL (multiple-instance learning), SVM (support vector machine), and MCS (multiple classifier systems) results are presented. The proposed system of MLeMIL for the SVM is 97.8% efficient, and image classification for the MCS is 95% efficient [33]. The existing MIL system for automated image indexing is 89% efficient, and hyper-spectrum image classification is 72% efficient. Classification is an ordered set of related categories used to group data according to similarities. It consists of codes and descriptors and allows survey responses to be put into meaningful categories to produce valuable data.

### 4.2. The Standard Error

The standard error of the estimate represents the difference between actual data values and those anticipated by the given model or the average deviation of the errors. A sample’s effectiveness, accuracy, and consistency are calculated using the standard error [34]. The estimated standard deviation of a statistical sample population is known as a statistic’s standard error (SE). The standard error is equal to σ divided by $\sqrt{n}$, where σ represents the standard deviation and $\sqrt{n}$ Represents the square root of the data’s sample count (as expressed in Equation (13)).

(13) $$Standard\;error=\frac{\sigma }{\sqrt{n}}$$

Virtual image sensing involves an electronic device that converts an optical image into an electronic signal. It is used in digital cameras and imaging devices to convert the light received by the camera or imaging device lens into a digital image. Table 3 shows the relationships in virtual image sensing based on the data errors. The standard error is calculated by dividing the standard deviation by the number of image sample square roots. Virtual image sensing is based on the data error inference result [35]. The image count was 15 to 650, and the increased error standard deviation accuracy range was 24%.

**Figure 11:**
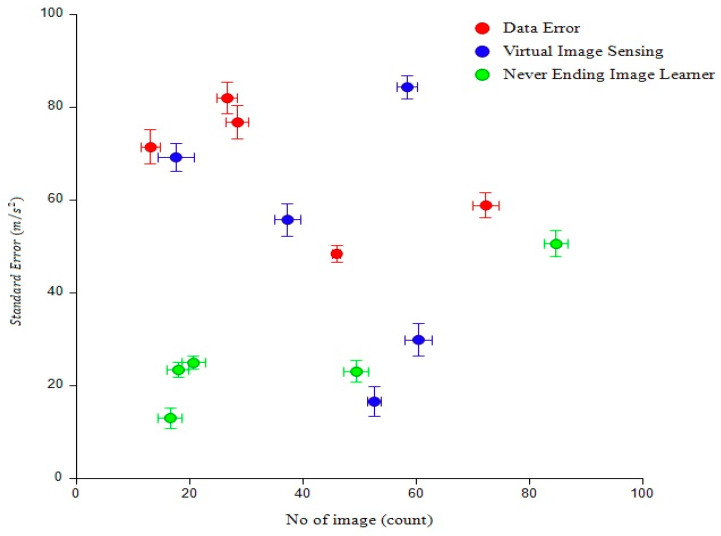
Analysis of standard data error.

Figure 11 shows that graph comparison compared to data error was 83% efficient, virtual image sensing was 85% efficient, and never-ending image learning was 54% efficient. The result for the proposed method was 24% efficiency. This graphical representation is based on the number of images and error standard deviations. A data error occurs when a datum in a digital medium has been altered erroneously. Such errors can be manifested as several incorrect bits or even a single bit that is 0 when it should be 1 or vice versa [30].

### 4.3. Time Duration and Normalized Frequency

The normalized frequency equals one divided by the number of image samples (Equation (14)). Table 4 shows common disease attribute diagnoses based on time duration and normalized frequency; the duration of an event or state is the time in which it happens or exists.

(14) $$Normalized\;frequency=\frac{1}{No\;of\;image\;sample}$$

Normalized frequency (f) is a quantity with a frequency dimension. You need only divide the frequency between counts by the number of image samples. A disease’s common attribute diagnosis is based on the time duration and normalized frequency inference result. The time duration was 0.67 to 12.43, and the increased normalized frequency accuracy range was 15%.

**Figure 12:**
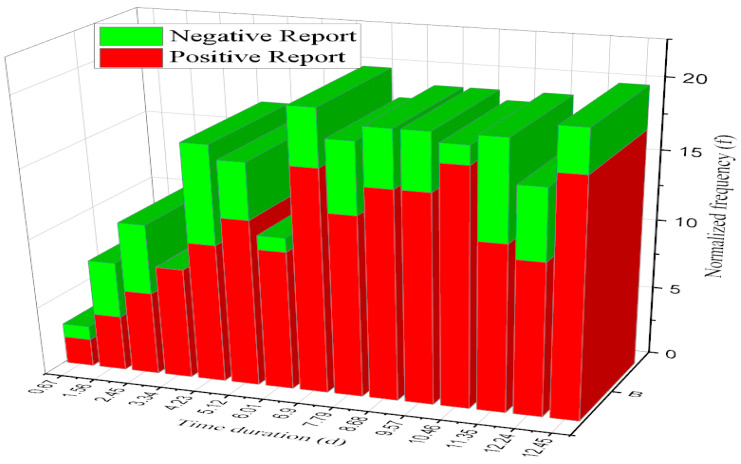
Analysis of common disease attribute diagnosis.

A health attribute is one component of a complete health state, representing one selected area of functioning that usually ranges from average to severely limited. A common attribute is a data element associated with a record in the system [36]. Figure 12 shows that a common attribute diagnosis is an illness or sickness characterized by specific signs or symptoms depending on the disease. This graph comparison result was compared with time duration and normalized frequency, producing an approximate result of 11.22% efficiency.

### 4.4. Predictions of Intelligent Analysis of Disease Factors

Results for the predictions of intelligent analysis of disease factors based on virtual image sensing are listed in Table 5. Virtual image sensing is software that, given the available information, processes what a physical sensor otherwise would.

This system learns to interpret the relationships between different variables and observes readings from different instruments.

Regarding the predictions of intelligent analysis of disease factors based on virtual image-sensing inference results, the image counts were from 12 to 156, and the increased prediction analysis accuracy range was 90.6%.

**Figure 13:**
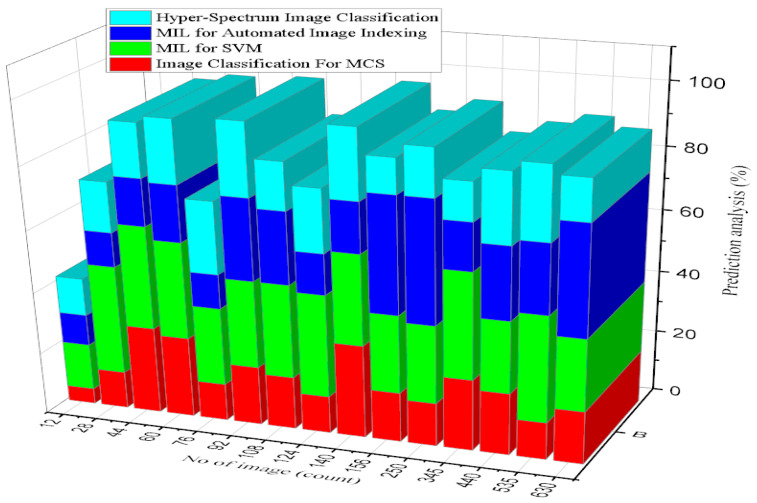
Prediction of intelligent analysis of disease factor analysis.

An intelligent health prediction system is intended to assist health professionals in their decision-making regarding medical situations. This system will provide the guidance and information needed for doctors. Figure 13 presents the prediction results of intelligent analysis of the disease defined [37]. In the clustering graph comparison results, the proposed system of MIL for SVM was found to be 45% efficient, and image classification for MCS was 20% efficient. The existing system of MIL for automated image indexing was 28% efficient, and hyper-spectrum image classification was 18% efficient.

### 4.5. Image Classification for Prediction Functions

Measurements of classification accuracy provide a categorical basis for diagnosing the a priori undefined data composition and the core of the data classes. Consequently, placing a single item in one of the selected classes achieved the highest classification accuracy score within the ideal data range of 70% to 90%. This finding may also be factual for virtual image-sensing data. According to automated prediction, the broad phrase “Online healthcare system Classification accuracy” defines how closely a measurement resembles the actual value [14]. Random and systematic mistakes occur when the phrase is used to describe collections of classification accuracy measurements made for the exact measurement. This classification accuracy allows the correct generation of data quality features from real-world expected data. The information is accurate and error-free thanks to this classification system’s correctness. The accuracy of classification data is based on hospital medical records. Since better classification accuracy metrics depend on correctness, the information must be accurate. The image classification for predicting inference results shows that the image classification level is 2 to 128 and that the increased predicting function accuracy range is 62.4%.

In statistics and machine learning, a linear predictor function is a linear function of a set of coefficients and explanatory variables whose value is used to predict the outcome of a dependent variable. An image is selected and loaded to predict image classification and resized to a predefined size, such as y (y pixels) [15]. Then, the pixel value is scaled to the range [0,y] and selected, the pre-trained model is run, and the results are displayed. The image classification prediction function experiment results are shown in Table 6.

**Figure 14:**
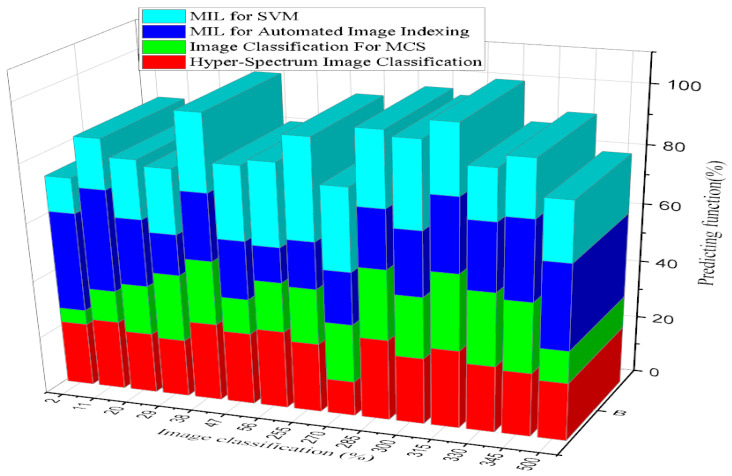
Analysis of Image Classification Against Prediction function.

According to Figure 14, showing the clustering graph comparison results, the proposed system of MIL for SVM was found to be 75% efficient, and image classification for MCS was 40% efficient. The existing system of MIL for automated image indexing was 60% efficient, and hyper-spectrum image classification was 33% efficient. Image classification refers to extracting information classes from a multiband raster image. The main objective of image classification is to group all the pixels in a particular image into a specific smart analysis of disease factors with the virtual image-sensing cover class. The resulting raster from image classification can be used to create thematic maps [20].

## 5. Conclusions

Never-Ending Image Learner to forecast illness variables allows structural risk reduction and chooses from finite data images using machine learning-enabled M-theory with virtual sensing to predict real-time images. Our findings could stimulate further research in deep learning and promote the idea of using machine learning for automatic online prediction of common disease attributes using Never-Ending Image Learner. The proposed method uses isotropic positioning and the working principles of never-ending image learning for image prediction. Classifications were performed and achieved an efficiency score of 96.54%; the clustering analysis was implemented and achieved a score of 95.22%; common disease attribute diagnosis was performed and achieved an efficiency of 86.64%, and the predictions of smart analysis concerning disease factors had a 6.54% higher value. This study achieved a higher accuracy level of 93.11%, a reliability of 94.35%, and a specificity of 94.68% compared to the existing methods. In the future, automatic online prediction of disease-common attributes can be found by comparing machine learning instead of machine learning and non-physical processes such as the business-oriented model. For this, we can use IoT-recommended Smart analysis of disease processes instead of Never Ending Image Learner. Machine Learning and Never Ending Image Learning, and Virtual image sensing used in this way can deliver a certain amount of Multi-access edge computing very well.

## Figures and Tables

**Table 1:** Use Never-Ending Image Learner (NEIL) for image-based prediction of diseases and symptoms.

SI No.	Disease	Symptoms
1	Alzheimer’s	Memory loss
		Poor judgment
		Confusion
2	Parkinson’s	Poor judgment
		Trouble speaking
3	Stroke	Slowed movement
		Trouble speaking

**Table 2:** Machine learning for automatic online prediction. Analysis of sample images and image-sensing accuracy.

Automatic Online Prediction Analysis		Image-Sensing Accuracy Clustering Analysis	
Sample Images (Count)	Image-Sensing Accuracy (%)	No Image Sample (Count)	Image-Sensing Accuracy (%)
25	66.7	10	66.6
58	76.5	28	59.09
70	78.05	46	56.25
85	78.21	64	54.76
150	77.22	64	53.84
235	80.67	64	53.22
286	80.32	64	52.7
350	83.1	136	52.43
400	86.6	154	52.17
500	88.3	172	51.96
650	85.6	190	51.78
725	87.9	208	55.5
815	92.8	226	59.4
900	95.6	244	60.78
950	97.8	262	65.34

**Table 3:** Analysis of virtual image sensing based on the standard data errors concerning standard deviations.

No. of Image Samples (Count)	Standard Deviation	Data Sample Count	Error STD (m/s^2^)
15	3.93	1	3.93
28	4.15	3	1.38
41	4.37	9	0.48
54	4.59	13	0.35
67	4.81	17	0.28
80	5.03	21	2.56
93	5.25	25	1.21
150	5.47	29	1.18
175	5.69	33	1.17
250	5.91	37	2.15
300	6.13	41	3.14
450	6.35	45	0.14
475	6.57	49	2.13
500	6.79	53	1.12
650	7.01	57	3.12

**Table 4:** Analysis of disease based on time duration and normalized frequency.

Time Duration (d)	No. of Image Samples (Count)	Normalized frequency (f)
0.67	5	0.23
1.56	18	0.058
2.45	31	0.032
3.34	44	0.028
4.23	57	0.0189
5.12	70	0.981
6.01	83	0.125
6.9	96	0.523
7.79	109	0.125
8.68	122	0.523
9.57	135	0.921
10.46	148	1.319
11.35	161	1.717
12.24	174	2.115
12.43	187	2.513

**Table 5:** Predictions of intelligent analysis of disease factors.

No. of Image Samples (Count)	Prediction Analysis (%)	No. of Image Samples (Count)	Prediction Analysis (%)
12	56.7	250	65.4
28	58.9	345	68.2
44	61.1	440	71
60	63.3	535	73.8
76	65.5	630	76.6
92	67.7	725	79.4
108	69.9	820	82.2
124	72.1	915	85
140	74.3	950	87.8
156	76.5	1000	90.6

**Table 6:** Analysis of image classification for prediction function.

Image Classification	Prediction Function	Image Classification	Prediction Function
2	45.3	135	56.8
11	47.9	150	57.2
20	50.5	165	57.6
29	53.1	180	58
38	55.7	195	58.4
47	58.3	210	58.8
56	60.9	225	59.2
65	63.5	240	59.6
74	66.1	255	60
83	68.7	270	60.4
92	71.3	285	60.8
101	73.9	300	61.2
110	76.5	315	61.6
119	79.1	330	62
128	81.7	345	62.4

## Data Availability

Data available on request from first author.
